# Multiplexed target enrichment of coding and non-coding transcriptomes enables studying *Candida* spp. infections from human derived samples

**DOI:** 10.3389/fcimb.2023.1093178

**Published:** 2023-01-24

**Authors:** Hrant Hovhannisyan, Antonio Rodríguez, Ester Saus, Mario Vaneechoutte, Toni Gabaldón

**Affiliations:** ^1^ Life Sciences Department, Barcelona Supercomputing Center (BSC), Barcelona, Spain; ^2^ Mechanisms of Disease Department, Institute for Research in Biomedicine (IRB), Barcelona, Spain; ^3^ Laboratory Bacteriology Research, Department of Diagnostic Sciences, Faculty of Medicine and Health Sciences, Ghent University, Ghent, Belgium; ^4^ Department of Biomedicine and Life Sciences, Universitat Pompeu Fabra (UPF), Barcelona, Spain; ^5^ Institució Catalana de Recerca i Estudis Avançats (ICREA), Passeig Lluís Companys 23, Barcelona, Spain

**Keywords:** *Candida*, host-pathogen interactions *in vivo*, RNA-Seq, probe-based enrichment, long non-coding RNA (IncRNA)

## Abstract

The study of transcriptomic interactions between host and pathogens in *in vivo* conditions is challenged by the low relative amounts of the pathogen RNA. Yeast opportunistic pathogens of the genus *Candida* can cause life-threatening systemic infections in immunocompromised patients, and are of growing medical concern. Four phylogenetically diverse species account for over 90% of *Candida* infections, and their specific interactions with various human tissues are still poorly understood. To enable *in vivo* transcriptomic analysis in these species, we designed and validated pan-*Candida* target capture probes to enrich protein-coding and non-coding transcriptomes. The probe-based enrichment approach outperformed enrichment based on differential lysis of host cells, and showed similar enrichment performance as an existing capture design, yet achieving better fidelity of expression levels, enabling species multiplexing and capturing of lncRNAs. In addition, we show that our probe-based enrichment strategy allows robust genotype-based identification of the infecting strain present in the sample.

## Introduction

Human fungal pathogens pose a serious global healthcare problem. The incidence of fungal infections, in their various forms, has increased over the last decade (Oren and Paul, 2014), currently affecting 25% of the global population and causing 1.5 million deaths every year (Havlickova et al., 2008; Bongomin et al., 2017). *Candida* yeasts are the most common cause of invasive fungal infections (Neofytos et al., 2013; Guinea, 2014). Current challenges to overcome *Candida* infections include the difficulty of accurate diagnosis and the emergence of novel pathogenic species (Satoh et al., 2009; Papon et al., 2013; Gabaldón et al., 2016; Consortium OPATHY and Gabaldón, 2019). Additionally, there are limited therapeutic options and these are losing efficiency due to the high capacity of adaptation to antifungal drugs shown by some *Candida* species (Cortegiani et al., 2018; Ksiezopolska and Gabaldón, 2018).

There are over 30 different *Candida* species that can infect humans (Papon et al., 2013). However, more than 90% of the infections are caused by the four species with the largest global incidence: *Candida albicans*, *Candida glabrata*, *Candida parapsilosis* and *Candida tropicalis* (Guinea, 2014). Despite their common genus name, these yeasts are phylogenetically diverse and have close non-pathogenic relatives, which suggests that their pathogenicity towards humans has emerged independently (Gabaldón et al., 2016). Given the probably different characteristics of their virulence, there is a need to better understand infection mechanisms and host-pathogen interactions specifically for each of these diverse species. However, the majority of studies so far have focused on *C. albicans* (Mayer et al., 2013), with research on non-albicans species significantly lagging behind (Hovhannisyan and Gabaldón, 2019; Kämmer et al., 2020; Pekmezovic et al., 2021). As a result, we still have a very poor understanding of how virulence and host-pathogen interplay vary across *Candida* pathogens.

Considering that host-fungus interactions comprise mutual adaptive processes involving transcriptional changes in both organisms, studies addressing the simultaneous regulation and expression of both host and pathogen genes during infection are most informative. Recent advances in Next Generation Sequencing (NGS) allow studying molecular interactions by assessing transcriptome dynamics of host and pathogen simultaneously (Amorim-Vaz and Sanglard, 2015; Enguita et al., 2016; Westermann and Vogel, 2018; Wolf et al., 2018; Hovhannisyan and Gabaldón, 2019). In this regard, high throughput transcriptome sequencing (RNA-Seq) has proven to be a powerful method for disentangling molecular interactions between human and *Candida* pathogens (Amorim-Vaz et al., 2015; Bruno et al., 2015; Liu et al., 2015; Rasheed et al., 2018). However, a major limitation of sequencing-based transcriptomic approaches, particularly when used *in vivo*, is the very low ratio of fungal/host RNA, which severely limits downstream analyses. For instance, in a recent RNA-Seq study using a murine model of vaginal candidiasis, the fungal reads represented less than 0.1% of the total reads (Bruno et al., 2015). This limitation can be bypassed in host-pathogen interaction studies by using *in vitro* experiments or animal models, whereby fungal loads can be controlled (MacCallum, 2012; Segal and Frenkel, 2018). In fact, the majority of studies investigating pathogenicity mechanisms of *Candida* pathogens have been performed *in vitro* (Enjalbert et al., 2003; García-Sánchez et al., 2004; Pekmezovic et al., 2021). However, it has been shown that, for example, *C. albicans* displays different transcriptional dynamics *in vivo* and *in vitro*, indicating that *in vitro* conditions only partially reflect real infections (Xu et al., 2015). Furthermore, while animal models (especially mammals) better resemble the human host, there are economic and ethical issues precluding their extensive use.

There are two main approaches for overcoming the low proportion of fungal RNA encountered *in vivo*. First, fungal RNA enrichment can be attained by selectively lysing human cells, chemically or mechanically, followed by centrifugation, to discard human RNA (Andes et al., 2005; Rodríguez et al., 2019). This method takes advantage of the superior resistance of fungal cell walls as compared to mammalian membranes (Beauvais and Latgé, 2018). However, traditional lytic approaches required incubation with triton X-100 and DNase at 37°C in which transcriptomic profiles may have changed (Andes et al., 2005). Another more recent study used a specific lytic treatment with Buffer RLT to lyse human cells without incubation, and showed that *C. albicans* cells were efficiently retained from a mixture with human cells, while enriching fungal genetic material (ITS) with high quality (Rodríguez et al., 2019). However, that study did not evaluate the applicability of the host cell lysis strategy for RNA-Seq analysis. A second approach consists of selecting fungal RNA/DNA molecules using oligonucleotide probes that specifically bind to fungal sequences. In this regard, SureSelect probe-based enrichment technology has been applied to enrich *C. albicans* (Amorim-Vaz et al., 2015) and *Candida glabrata* (Schrevens et al., 2022) transcripts from *Galleria mellonella* and murine infection models to subsequently perform RNA-Seq. These studies showed that probe-based enrichment increased fungal RNA relative abundance up to 2000-fold, while only significantly altering the expression levels of ~3-3.6% of the genes. This technology was also successfully used on a mouse model of aspergillosis (Chung et al., 2018).

Here, we designed a novel target-capture enrichment approach based on SeqCap (Roche) technology that improves over the previously existing enrichment designs in two main ways. Firstly, in addition to protein-coding genes, our probe set also targets long non-coding RNAs, enabling the analysis of these poorly studied molecules. Secondly, it combines targets for the full transcriptomes (*i.e*. coding and non-coding) of the four main *Candida* pathogens, expanding its use for comparative transcriptomic analyses of different species, the study of co-infections, and the direct study of clinical specimens from the majority of cases of candidiasis. We tested the efficacy of our enrichment approach and the accuracy of downstream RNA-Seq analyses by using human vaginal swab samples spiked with defined amounts of *C. albicans* cells. Additionally, we compared the results of probe-based enrichment with one of the aforementioned enrichment methods based on selective lysis of human cells (Rodríguez et al., 2019). Our results indicate that our approach efficiently enriched fungal RNAs and did so to a significantly higher level as compared with the differential lysis approach. Most importantly, the probe-based enrichment did not significantly alter expression levels, with only ~0.3-1.5% of the fungal genes being affected. Moreover, we show that besides standard RNA-Seq analyses such as transcriptome profiling and differential gene expression, the probe-based enrichment results can serve to perform additional analyses such as variant calling of the infecting strain.

## Materials and methods

### Preparation of spiked-in vaginal samples

A total of 48 *Candida*-negative vaginal swabs (E-Swabs, COPAN Diagnostics, CA, USA), from 48 premenopausal, non-pregnant and healthy women of at least 18 years, were collected at the University Hospital of Ghent (Ghent, Belgium, informed consent was obtained from all participants. Approval ethical committee EC/2016/0192). Upon collection, the samples were immediately immersed in 1.2 ml RNAlater (ThermoFisher Scientific, Waltham, MA) and stored first at 4 °C overnight and then at -80 °C. Absence of *Candida* was tested by a) culture in Sabouraud glucose agar with chloramphenicol (Merck KGaA) and subsequent species screening with MALDI-TOF analysis, b) microscopic visualization through wet mount in combination with phase contrast microscopy and c) Gram-staining in combination with light microscopy.

For subsequent analysis, the stored vaginal samples were thawed at 37°C and briefly vortexed. Swabs were then discarded and the RNAlater solutions containing the cells were all pooled together in a 100 ml falcon tube and mixed by pipetting. Then, two aliquots of 12.5 ml of the vaginal samples pool were spiked with *C. albicans* SC5314 cells to reach a final concentration of 10^5^ cells/ml, and 10^3^ cells/ml respectively, and then split in 1-ml aliquots and stored at -80°C until RNA extraction. Prior to preparation of spike-in samples, *C. albicans* had been incubated at 32°C in Sabouraud plates overnight, whereafter a colony was incubated in YPD broth at 32°C, with shaking, overnight. Fungal cells were counted with a microscope by using a hemocytometer (Neubauer chamber).

### Lytic enrichment with buffer RLT + β-mercaptoethanol

Because the use of buffer RLT + β-mercaptoethanol enrichment (treatment “B”) might change gene expression, which would no longer match the transcriptional profile encountered during the infection, we did not only test the efficiency of the probe-based enrichment in vaginal samples but also checked whether gene expression is affected during the enrichment process. To this purpose, we included a control in which RNA extraction was performed directly from the vaginal samples without a preceding lytic enrichment. In addition, we also included a lytic enrichment with pre-treatment with thiolutin (treatment “BT”), a known inhibitor of transcription (Jimenez et al., 1973; Tipper, 1973; Kebaara et al., 2006; Pelechano and Pérez-Ortín, 2008) to prevent gene expression changes. Finally, we also used thiolutin without lytic enrichment to test whether the thiolutin modified gene expression (treatment “T”). In summary, four different treatments were tested: 1) No thiolutin and no lytic enrichment (Treatment “N”); 2) Thiolutin and no lytic enrichment (Treatment “T”); 3) No thiolutin and buffer RLT + β-mercaptoethanol lytic enrichment (Treatment “B”); 4) Thiolutin and Buffer RLT + β-mercaptoethanol lytic enrichment (Treatment “BT”).

All treatments were performed in triplicate, starting from 1-ml aliquots, which had been stored at -80 °C, of a pool of vaginal samples spiked with either 10^5^ or 10^3^ *Candida* cells. One-ml aliquots were thawed and centrifuged at maximum speed (> 20 000 g) for 5 min in a benchtop centrifuge to collect human and fungal cells and to discard RNAlater. Pellets of cells were then used for treatments N, T, B or BT. A total of six vaginal samples, three containing 10^5^ *Candida* cells (hereafter “High fungal load”) and three with 10^3^ *Candida* cells (hereafter “Low fungal load”), were used for each different treatment. Each sample was processed with the respective treatment prior to RNA extraction. To minimize handling time and post-sampling changes in expression and RNA degradation, only six samples were processed at a time. The following treatments were applied: For treatment N, pellets containing human and fungal cells were directly used for RNA extraction with the RiboPure Yeast Kit. For treatment T, pellets of human and fungal cells were resuspended in 200 µl ice-cold PBS containing thiolutin at a final concentration of 20 µg/ml and incubated on ice for 15 min to stop transcription events. Pellets of cells after centrifugation at 20 000 g for 8 min, at 4°C, were directly used for RNA extraction. For treatment B, pellets of human and fungal cells were resuspended in 600 µl of RLT buffer containing 1% β-mercaptoethanol (*i.e.*, 143 mM) and pipetting up and down to lyse human cells. Samples were centrifuged at max speed (> 20 000 g) for 8 min, at 4°C, to collect intact yeast cells. Supernatants containing cell debris and nucleic acids from human cells were carefully discarded without disturbing the fungal cell pellet.

For treatment BT, pellets of human and fungal cells were resuspended in 200 μl ice-cold PBS containing thiolutin at a final concentration of 20 μg/ml. Samples were incubated for 15 min on ice to stop transcription events. Then, samples were centrifuged at 20 000 g for 8 min, at 4°C, to collect the cells. Human cells were lysed with RLT buffer and samples were again centrifuged to collect intact yeast cells, as in treatment B. Yeast cells were finally subjected to RNA extraction with the RiboPure Yeast Kit (ThermoFisher Scientific), following manufacturer’s specifications.

### Custom design of oligonucleotide probe-based targeted enrichment

We designed a custom pan-*Candida* enrichment kit for SeqCap technology (Roche, Basel, Switzerland). Our design included probes targeting whole transcriptomes of the four most widespread *Candida* pathogens - C*. albicans*, *C. glabrata*, *C. parapsilosis* and *C. tropicalis*, including all previously annotated features, such as protein-coding genes and non-coding RNAs, and newly annotated long non-coding RNAs (lncRNAs), which were predicted in our study (see below). For obtaining annotated features, we first fetched the corresponding reference genomes and genome annotations for each species: *C. albicans* SC5314 strain (assembly 22), *C. glabrata* CBS138, *C. parapsilosis* CDC317 and *C. tropicalis* MYA-3404 from the *Candida* Genome Database (last accessed in July 2017) (Skrzypek et al., 2017). Considering that the genome of *C. albicans* is phased, we used only haplotype A for all downstream analyses. For each species, we extracted transcriptomes from genomes and genome annotations using the *getfasta* function of bedtools v. 2.26.0 (Quinlan, 2014).

We retrieved a predicted set of *C. parapsilosis* lncRNAs (n=618) from (Thuer, 2017). For the remaining species, we performed *de novo* prediction of lncRNAs. For this, we used all publicly available RNA-Seq datasets for these species available at the Sequence Read Archive (SRA) database as of June 2017 (Leinonen et al., 2011). This comprised a total of 69 samples for *C. albicans*, 39 samples for *C. glabrata* and 36 samples for *C. tropicalis* (list of used sample accession numbers is available in **Table S1**). Reads were mapped against the corresponding reference genome using TopHat2 v. 2.1.1 (Kim et al., 2013). We then performed reference guided transcriptome assembly using Cufflinks v. 2.2.1 (Trapnell et al., 2012), and, for each species, merged individual assemblies into a unified assembly and compared it with reference annotations. Subsequently, we selected novel intergenic transcripts longer than 200 base pairs and assessed their coding potential using CPC v. 0.9 software (Kong et al., 2007). Transcripts with no coding potential were considered as lncRNAs. These analyses resulted in 187, 93 and 485 putative lncRNAs for *C. albicans*, *C. glabrata* and *C. tropicalis*, respectively.

Additionally, we included the sequences of the External RNA Controls Consortium (*i.e*. ERCC) spike-in RNA control molecules to our probe design. Once all necessary sequences were obtained, we used a custom python script to design probes targeting these sequences (Iraola-Guzmán et al., 2020). This script designs probes with variable length (ranges between 55-70 bps), optimizing GC content, transcript coverage and number of probes per transcript. The custom probes were subsequently ordered from Roche and received as a SeqCap RNA Developer Enrichment Kit.

### Library preparation

Sequencing libraries were prepared using the TruSeq Stranded mRNA Sample Prep Kit v2 (ref. RS-122-2101/2, Illumina) according to the manufacturer’s protocol for all samples. All reagents subsequently mentioned are from the TruSeq Stranded mRNA Sample Prep Kit v2, unless specified otherwise. For each sample, 500 ng of total RNA were used for poly(A)-mRNA selection using streptavidin-coated magnetic beads. Briefly, all samples were subsequently fragmented to approximately 300 bp. cDNA was synthesized using reverse transcriptase (SuperScript II, Invitrogen) and random primers. The second strand of the cDNA incorporated dUTP in place of dTTP. Double-stranded DNA was further used for library preparation. dsDNA was subjected to A-tailing and ligation of the barcoded Truseq adapters. All purification steps were performed using AMPure XP Beads (Agencourt). Library amplification was performed by PCR on the size-selected fragments using the primer cocktail supplied in the kit. Final libraries were analyzed using Agilent DNA 1000 chip (Agilent) to estimate the quantity and check the size distribution, and were then quantified by qPCR using the KAPA Library Quantification Kit (KapaBiosystems) prior to amplification with Illumina’s cBot.

Prior to sequencing, 15 µl of each library was used to perform fungal RNA enrichment using our custom SeqCap RNA Developer Enrichment Kit (see above), following the manufacturer’s instructions. Briefly, we first prepared the multiplex cDNA sample library pool (a mixture of all libraries), which was mixed together with 5 μg of COT Human DNA and 2,000 pmol of the corresponding multiplex hybridization-enhancing oligo pool (to prevent hybridization between adapter sequences). After drying this mixture in a DNA vacuum concentrator at 60°C, the following reagents were added: 7.5 μl of 2X Hybridization Buffer and 3 μl of Hybridization Component A. Samples were vortexed for 10 seconds, centrifuged at maximum speed for 10 seconds, and then left at 95 °C for 10 min to denature the cDNA. After a short centrifugation at maximum speed for 10 seconds, the mixture was transferred to a 4.5 μl aliquot of SeqCap RNA probe pool previously prepared in a 0.2 ml PCR tube, vortexed for 3 seconds and centrifuged at maximum speed for 10 seconds more. Finally, the mixture was incubated in a thermocycler at 47°C for 20 hours (with the thermocycler lid set at 57°C). After the hybridization step, the sample was washed and the captured multiplex cDNA sample was recovered from the mixture with SeqCap streptavidin beads, and amplified following the manufacturer’s instructions. PCR products were purified with AMPure XP Beads (Beckman Coulter).

The quality of the enriched pool was assessed with a Bioanalyzer DNA 1000 chip (Agilent). Non-enriched libraries and the enriched pool of libraries were loaded and sequenced using 2 x 125 read length on Illumina’s HiSeq 2500.

### RNA-Seq data analysis

We performed quality control of raw sequencing data using FastQC v. 0.11.6 (http://www.bioinformatics.babraham.ac.uk/projects/fastqc) and Multiqc v. 1.0 (Ewels et al., 2016). Samples having bases with low quality or traces of adapter sequences were filtered using Trimmomatic v. 0.36 with TruSeq3-PE-2 adapters with 2:30:10 options and minimum read length of 50 bps (Bolger et al., 2014). Reads passing quality control were mapped to the concatenated reference genomes of *C. albicans* SC5314 haplotype A (assembly 22) and primary human genome assembly GRCh38 obtained from Ensembl database (release 89, last accessed in June 2018) (Hunt et al., 2018). Read mapping was performed using splice-junction aware aligner STAR v. 2.5.2b (Dobin et al., 2013) using basic two-pass mode and default parameters. Read summarization was performed by STAR and Featurecounts v. 1.6.4 (Liao et al., 2014). The counting of reads mapped to human and yeast genomes was performed with a custom python script read_count.py v. 1 available at https://github.com/Gabaldonlab/Probe_enrichment. Previous studies have demonstrated only negligible cross-mapping rates (i.e. reads originating from one organism but mapping to another) between the RNA-Seq data *C. albicans* and human host (Hovhannisyan et al., 2020; Pekmezovic et al., 2021). Hence the reads that mapped to the two genomes equally well were discarded from the analysis. The analysis of potential read cross-mapping of transcriptomes between the four studied species was performed using Crossmapper v. 1.1.1 (Hovhannisyan et al., 2020).

Differential gene expression analysis was performed using the DESeq2 v. 1.26 Bioconductor package (Love et al., 2014). Genes with |log2 fold change| > 2 were considered as differentially expressed (DE) as this was the criterion used in (Amorim-Vaz et al., 2015).

To compare the fold enrichments resulting from our probe design and from (Amorim-Vaz et al., 2015), we first modeled the dependency of fold enrichment from our initial fungal proportions on a log2 scale using the *lm* function of R v. 3.5.3. Subsequently, based on this model and using the *predict* function, we predicted the values of fold enrichment which could have been obtained with our probes if applied to the initial fungal proportions observed in (Amorim-Vaz et al., 2015).

### Variant calling

We used the bam files produced by STAR to perform variant calling using bcftools v. 1.6 with mpileup function, setting –max-depth 2000 option and default parameters. For downstream analysis we used variants with QUAL>20. To test whether these variants allow identification of the spiked *C. albicans* strain SC5314, we compared the variants called in our study with those of 58 strains representing the major clades of the *C. albicans* phylogeny (Ropars et al., 2018). Variant calling of those samples was done as described previously (Mixão and Gabaldón, 2020a). Considering that the data of previously published strains were generated on the basis of whole genome sequencing, we first performed variant subsetting with vcftools v0.1.16 of all vcf files to analyse only variants within genic regions, and then retained the variants with QUAL>20. Further, variants present in each of our samples with N treatment (*i.e*. without lytic enrichments and without thiolutin treatments) were compared with variants present in 58 C*. albicans* strains (Mixão and Gabaldón, 2020a) representative of *C. albicans* diversity using the bcftools *isec* function.

All other data analyses and visualizations were performed in R v. 3.5.3 using various packages.

RNA-Seq data generated in this study are accessible in Sequence Read Archive under the PRJNA721739 BioProject accession number.

The codes for reproducing the results of this study are available at https://github.com/Gabaldonlab/Probe_enrichment.

## Results

### Design of a pan-*Candida* probe-based enrichment kit targeting coding and non-coding transcriptomes

We set out to design and validate a probe-based enrichment approach suitable for the analysis of coding and non-coding transcriptomes of the four major *Candida* pathogens in samples with low content of fungal RNA, such as human clinical samples. To this purpose, we designed probes targeting all annotated genes in *C. albicans*, *C. glabrata*, *C. parapsilosis* and *C. tropicalis.* In addition, we predicted lncRNAs of these species and included probes for them in our design (see Materials and Methods). Our final dataset targets 24,282 previously annotated features, including protein coding genes and non-coding RNAs, and 1,383 newly predicted lncRNA genes from the four mentioned species.

Before proceeding with the synthesis of the probes, we first checked the rates of read cross-mapping that potentially can occur in two different scenarios – first, in the case of a dual RNA-Seq analysis comprising human and *C. albicans* reads, and second, in the case of a sample comprising more than one of the four studied *Candida* species. For the first scenario, a virtual absence of cross-mapping was already reported in our previous study of vaginal epithelial cells interacting with the four fungal species (Pekmezovic et al., 2021). For the seconds scenario, we performed an in-silico estimation of read cross-mapping between the transcriptomes of the studies species using Crossmapper software (Hovhannisyan et al., 2020). Crossmapper simulates reads from the supplied reference sequences (in this case, the transcriptomes of the four yeasts), and then simultaneously maps all the simulated data back to that references. Considering that simulated reads contain the exact information about species of origin, based on this information the software calculates the number and proportion of reads that originate from one species but map to a different one. The results of this in-silico analysis (**Data Sheet 1**) show that sequencing with paired-end reads of 50 bp length the cross-mapping of to wrong transcriptomes is nearly non-existent – with a maximum of ~2300 reads out of 20 million in the case of *C. albicans* and *C. parapsilosis*. This result indicates that even in the case of potential off-target binding of probes from one species to another, that kind of bias can be effectively resolved on the read mapping step of bioinformatics analysis.

We then used the SeqCap enrichment approach by Roche to synthesize probes, although our design could easily be adapted to other technologies. In brief, SeqCap is a hybridization-based technology, which uses biotinylated oligonucleotide probes that specifically bind to target sequences in NGS libraries of interest prior to sequencing. After target binding, probes are pulled by streptavidin-coated magnetic beads to obtain sequencing libraries, which are highly enriched with target regions.

### Targeted probe-based capturing efficiently enriches fungal transcripts and outperforms differential lysis approaches

**Figure 1:**
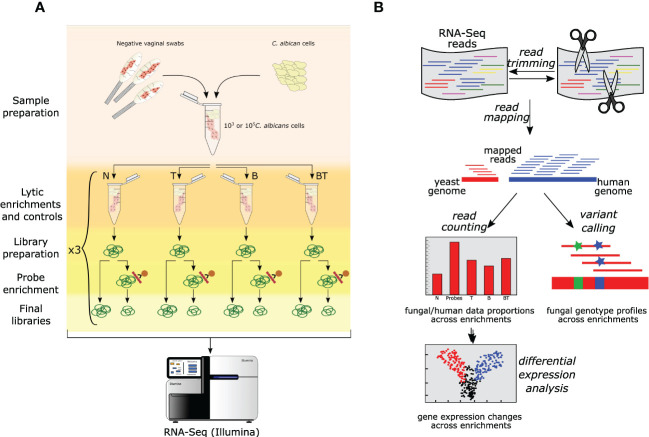
Overall experimental design and data analysis pipeline used in this study. **(A)** Schematic representation of the experimental setup. A pool of *Candida*-negative vaginal samples was spiked with different loads of *C*. *albicans* cells. Spiked samples further underwent different enrichments and sequencing. Different lytic enrichment approaches are: N - No thiolutin and no lytic enrichment; T - Thiolutin and no lytic enrichment; B - No thiolutin and buffer RLT + β-mercaptoethanol lytic enrichment; BT - Thiolutin and Buffer RLT + β-mercaptoethanol lytic enrichment. **(B)** Schematic RNA-Seq data analysis workflow employed in this study (see Materials and Methods for details).

To test our probe-based enrichment approach and compare it with the lytic enrichment, we used both approaches in parallel and in different combinations on a control sample consisting of a pool of vaginal swabs from healthy donors that was spiked with a known number of *C. albicans* cells - 10^3^ or 10^5^ (**Figure 1**, see **Materials and Methods**). Samples were non-treated (N) or incubated with thiolutin (T) or lytically enriched (B) or both incubated with thiolutin and lytically enriched (BT). Subsequently, all samples were subjected to RNA extraction and RNA-Seq library preparation. The libraries were then split into two equal parts, one of which was further enriched with SeqCap oligonucleotide probes. Finally, all libraries were subjected to RNA-Seq. It must be noted that our RNA-Seq library preparation included a poly-A selection step, and consequently the probes are not expected to enrich the non-polyadenylated transcripts, such as tRNAs, rRNAs and ITS. Nevertheless, the probe design includes those transcripts too, hence future studies could enrich those in libraries comprising total RNA.

**Figure 2:**
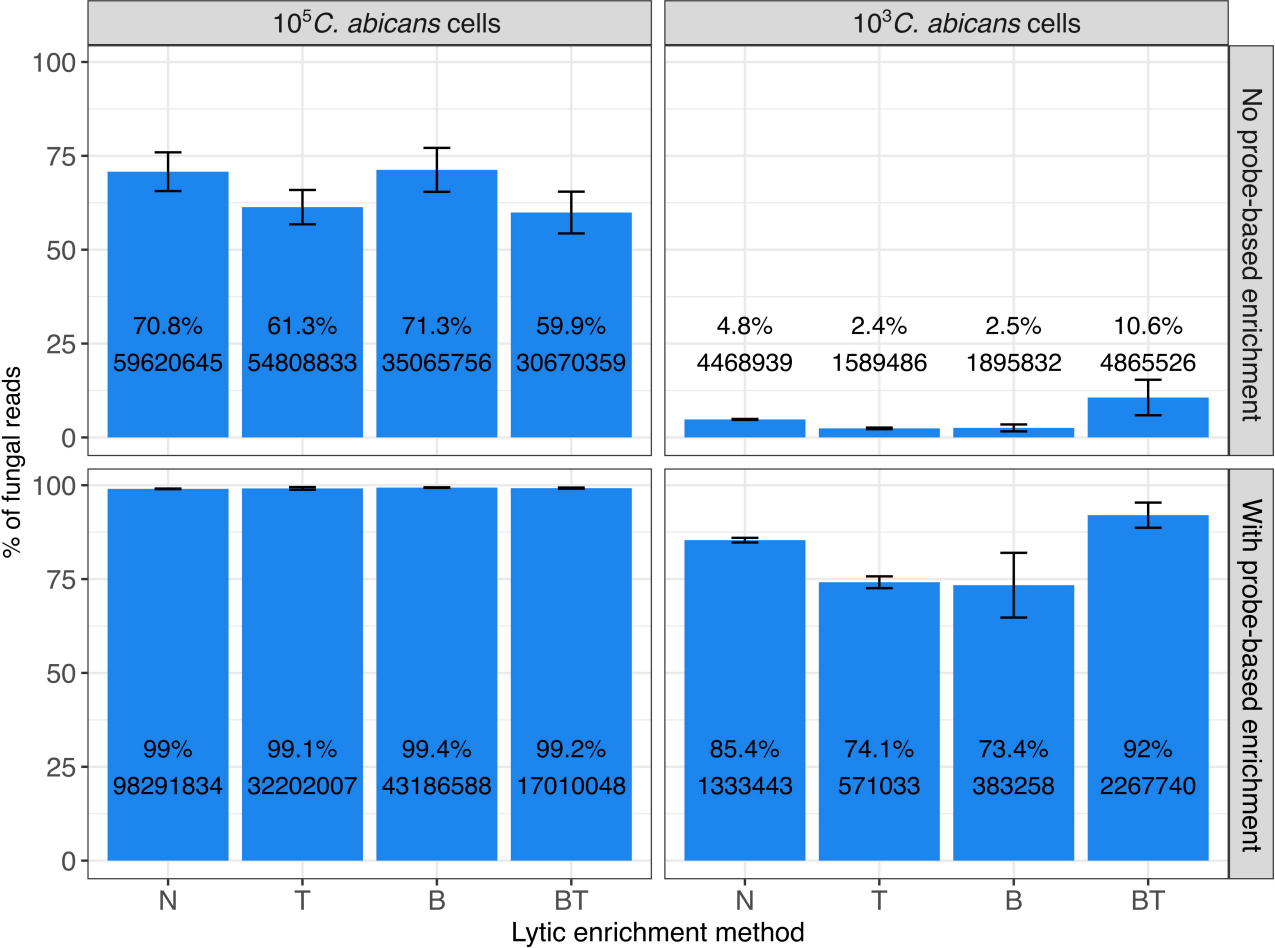
Proportions of fungal RNA reads obtained after different treatment/enrichment approaches (N, T, B, BT), and with or without probe-based enrichment, as determined by RNA-Seq. Left panel shows data of experiments with 10^5^ C*. albicans* cells (“High fungal load”), right panel - with 10^3^ fungal cells (“Low fungal load”). Upper panel shows data on experiments with no probe-based enrichment, and the bottom panel - with probe-based enrichment. N: No thiolutin and no lytic enrichment; T: Thiolutin and no lytic enrichment; B: No thiolutin and buffer RLT + β-mercaptoethanol lytic enrichment; BT: Thiolutin and Buffer RLT + β-mercaptoethanol lytic enrichment. Bars represent the proportion (in %, y axis) of mapped fungal reads over the total number of mapped reads to *C*. *albicans* and the human genomes, calculated as mean and standard deviation across three replicates. Labels on the bars represent the mean percentage (at the top) and mean raw read counts (at the bottom) across replicates.

After RNA-Seq, we mapped the data to the concatenated reference genomes of *C. albicans* and human, and calculated proportions of reads mapped to either species (**Figure 2**).

Our results show that our probe-based enrichment significantly increases the fraction of fungal reads as compared to non-enriched samples. Fold enrichment was proportionally higher in samples having lower initial amounts of fungal RNA - from 70.8 to 99% (1.4-fold enrichment) for 10^5^ fungal cells/ml and from 4.8% to 85.4% (~17-fold enrichment) for 10^3^ cells, on average across replicates of sample without lytic enrichments.

Furthermore, we also compared the probe-based enrichment approach with a lytic enrichment procedure based on the selective lysis of human cells with buffer RLT followed by centrifugation to discard contaminating human DNA and RNA, that had been shown previously to enrich fungal ITS (Rodríguez et al., 2019). However, in this study we found that the differential lysis approach did not enrich fungal RNA significantly when enrichment over the bulk transcriptome is assessed, with the exception of BT at low fungal load. Overall, our data suggest that targeted probe-based enrichment significantly outperforms the human cell lysis method in enriching fungal RNA from clinical samples.

We also assessed the efficiency of the enrichment strategies for the set of newly predicted lncRNAs (**Figure S1**). As expected, we obtained proportionally similar enrichment as for the whole dataset (**Figure 2**).

Further, we aimed to compare the efficacy of probe-based targeted enrichment with the results of the study of Amorim-Vaz et al. (Amorim-Vaz et al., 2015), which used SureSelect technology for enriching *C. albicans* transcriptome from animal models of infection. This study has reported significantly higher fold enrichment, ranging from 670 to 1670, than observed in our case (maximum of 43, **Figure S2A**). However, that study was performed using mouse and *Galleria mellonella* animal models, whereby the initial fungal proportions of 0.03%-0.1% were significantly lower than in our study. In fact, the highest initial fungal proportion observed in that study (0.1%) is 15-fold lower than the lowest proportion in our experiment (~1.48%). To make our results comparable, we modeled the dependency of fold enrichment on the initial fungal proportion. Then we predicted the fold enrichment values which potentially could be obtained with our probes if using the same initial proportions of fungal RNA as in the study of (Amorim-Vaz et al., 2015). The results of this modeling (**Figure S2**) show that the actual fold enrichment of our probes and of those used in the previous report are similar.

### High fidelity of gene expression levels after probe-based enrichment

**Figure 3:**
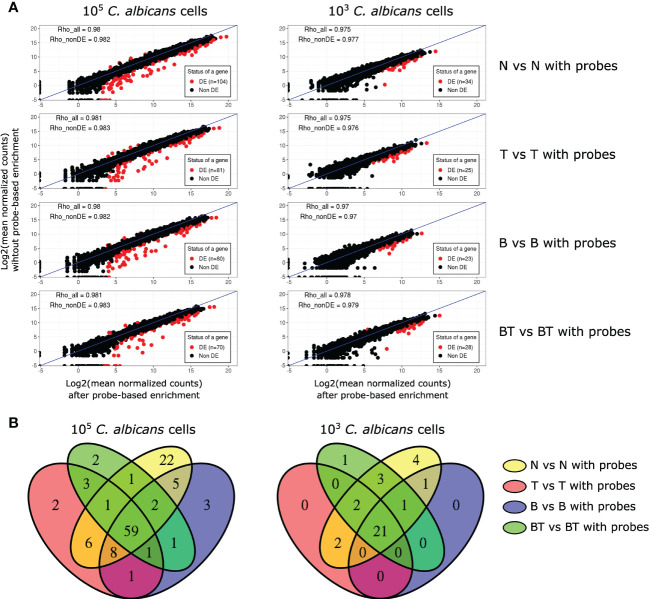
Analysis of the effect of probe-based enrichment on the expression levels of *C*. *albicans* genes across all experimental conditions. **(A)** Correlation plots of average normalized fungal read counts between original (y-axis) and probe-enriched (x-axis) samples. DE - Differentially Expressed; Rho_all - Spearman’s correlation coefficients calculated with all genes; Rho_nonDE: Spearman’s correlation coefficients calculated with all genes, excluding differentially expressed ones; **(B)** Venn diagrams of DE genes between non-probe-enriched and probe-enriched samples.

We further tested whether probe-based enrichment alters the expression levels of fungal genes, which could bias downstream analyses. First, we analyzed the mean normalized read count data of the same samples before and after probe-based enrichment (**Figure 3A**).

Differential gene expression analysis showed that probe-based enrichment significantly alters the expression of a small number of genes (n=23-104), constituting ~0.3-1.5% of the *C. albicans* transcriptome. The proportion of genes with altered expression in our study is lower than that reported in the study of Amorim-Vaz et al. (2015), *i.e*. ~3%. By re-analyzing the RNA-Seq data of that report and applying the same analysis approaches for the data of both studies, we observed that our targeted enrichment approach preserves the true expression levels more accurately (**Figure S3**).

Notably, most of the DE genes in our study have higher expression in non-probe enriched samples and are highly expressed, particularly in the samples with higher fungal load. These two observations suggest that the probes targeting these genes reach saturation. We further assessed whether genes with altered expression were randomly distributed or common between analysed conditions. Taking advantage of the fact that our experimental design allowed performing differential expression analysis between all pairs of original and probe-enriched samples in all tested conditions, we were able to show that most of the genes with biased expression are common between conditions (**Figure 3B**). This observation indicates that oligonucleotide probes systematically bias the expression levels of the same genes (see **Table S2**), allowing us to confidently identify and discard these genes from further analyses.

As expected (Jimenez et al., 1973), treatment with the transcriptional inhibitor thiolutin did not affect the expression levels of genes, except for 1 and 13 genes in the case of 10^3^ and 10^5^ C*. albicans* cells, respectively, which might be attributed to experimental manipulations.

### Probe-enriched RNA-Seq data allow variant calling analysis

RNA-Seq data can be used to call variants in the transcriptionally active parts of the genome. Hence, we further tested whether it is feasible to perform variant calling analysis using the probe-enriched data. For this, we performed SNP calling analysis for all datasets and compared the results between the probe-enriched and non-enriched samples (**Figure S4**). We observed that 50-75% of SNPs are retained after the probe enrichment. Interestingly, when analyzing the read depth (DP) parameter of variant calling results, which in essence indicate the sequencing depth of the variants, we observed that variants detected in both probe-enriched and non-enriched samples systematically had higher DP values than variants exclusively detected in non-enriched samples (**Figure S5**). This may indicate that variants identified exclusively in non-enriched samples are more likely to represent false-positive calls. This likely explains the lower amount of variants detected after probe-enrichment.

Conversely, we found that 80-90% SNPs that were identified after probe enrichment were identical to the ones obtained from the non-enriched samples.

We further tested whether the identified variants in our data are sufficient to determine the genetic background of the fungal cells that were spiked into the samples. To this end, we compared the variants identified in our samples with those present in a diverse set of 58 C*. albicans* strains (including SC5314), representing the major clades of the inter-strain phylogenetic tree of this species (Mixão and Gabaldón, 2020a).

**Figure 4:**
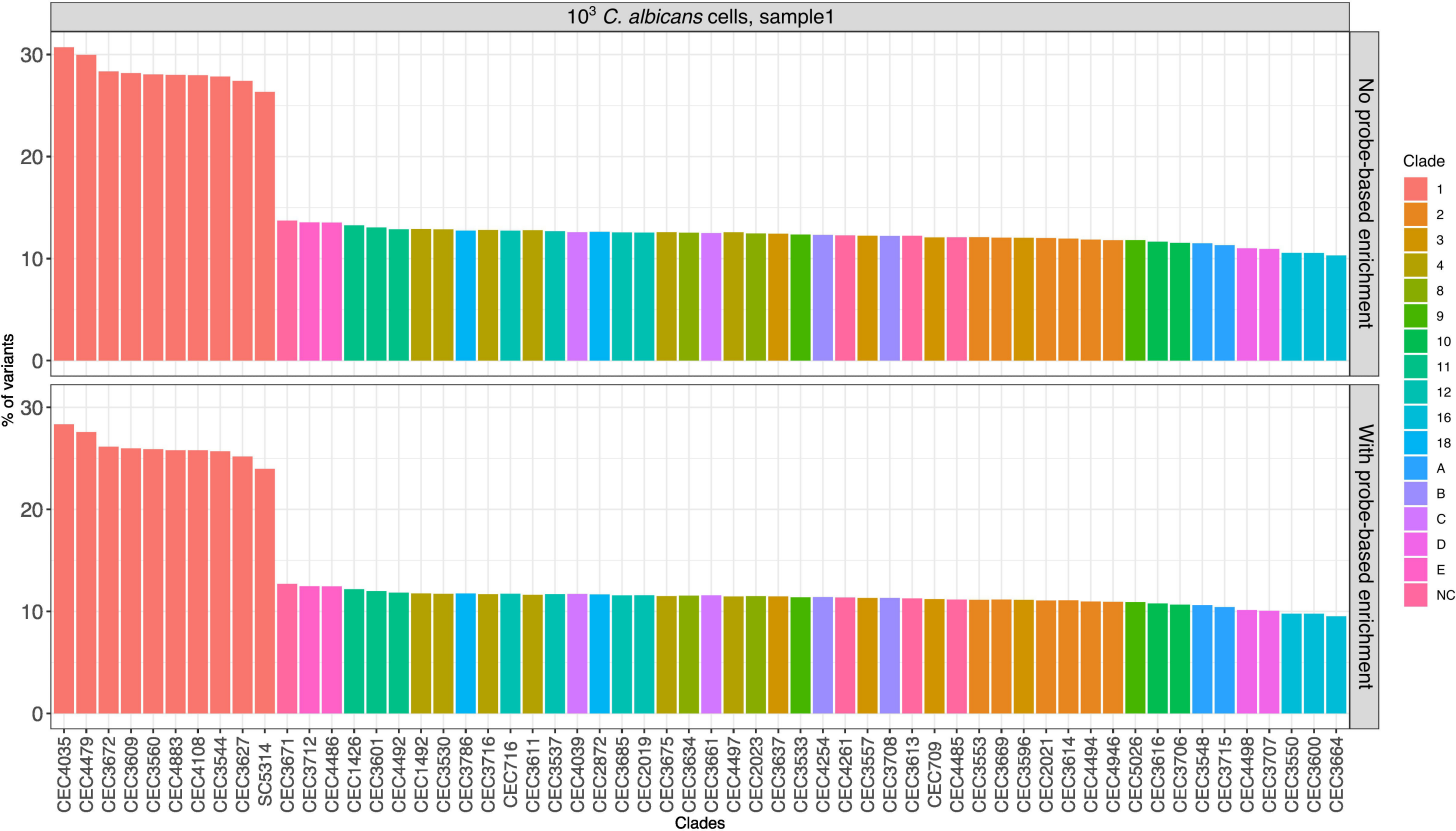
Comparison of variants of enriched and non-enriched sample (“N”) with previously published *C. albicans* strains. Each bar represents the proportion (in %) obtained by dividing the number of variants in common between the strain used in this study and a given published strain by the total number of variants of the published strain. Only the data of one sample from the 10^3^ fungal load are shown (see **Figure S6** for all samples from treatment “N”).

As a measure of genetic relatedness, we calculated the number of shared variants between our samples and any given strain, relative to the total number of variants in the latter. The results (**Figure 4**) of this analysis showed that the called variants before and after target enrichment provided sufficient resolution to identify the phylogenetic clade to which the strain belongs to, *i.e.* Clade 1, although not the specific strain (Ropars et al., 2018).

It must be noted that we observed similar results when using both enriched and non-enriched data, indicating that clade level identification, rather than identification of the specific strain, is due to a relatively limited resolution of RNA-Seq-based genotyping as such and not due to the probe-based enrichment.

Finally, using variant calling data we tested whether the probes of *C. albicans* SC5314 designed using its haplotype A also target the haplotype B. The two haplotypes have a divergence of 3.52%, or 35 variants per kilobase in heterozygous regions (Mixão et al., 2020b). To do this, we compared the distribution of homozygous and heterozygous variants in both probe-enriched and non-probe-enriched samples. If the probes would only select the haplotype A, then heterozygous variants will be lost when performing variant calling using the haplotype A as a reference. Our results (**Figure S7**) demonstrate that the distribution of homozygous and heterozygous variants is very similar between probe-enriched and non-probe-enriched samples, indicating that designed probes efficiently enrich both haplotypes of *C. albicans.* Importantly, our results also imply that the designed probes can also enrich transcriptomes of other *C. albicans* strains because average divergence of the same haplotype across strains (average of 6.7 variants per kilobase) is much smaller than the divergence between the two haplotypes (Mixão and Gabaldón, 2020a).

## Discussion

Human-*Candida* interaction studies that are performed *in vivo* are limited by the low proportion of fungal cells, a problem that is particularly important for transcriptome sequencing analyses. Here, we addressed this issue by designing a probe-based enrichment, targeting the complete coding and non-coding transcriptomes of four main *Candida* pathogens. We tested our design using a large-scale dual RNA-Seq experimental setup, allowing us to compare the efficiency of the probe-based enrichment with an alternative enrichment method, based on selective lysis of human cells (Rodríguez et al., 2019). We showed that the probe-based enrichment was significantly more efficient than the differential lysis approach. In addition, probe-based enrichment retained the relative abundance of transcripts, biasing the expression levels of only ~0.3-1.5% of the genes. Thus, this approach can be reliably used for standard downstream transcriptomics analyses such as the detection of differential gene expression. As for the differential human cell lysis approach, we observed a negligible enrichment achieved by this technique. A possible explanation of this result could be that the initial testing of these methods was based on measuring the fungal ribosomal ITS1 transcript, which in this study is removed by poly-A selection during the sequencing library preparation steps. This notion is confirmed by the fact that we did not detect any read mapping to ITS1 in any of our samples.

To our knowledge, there are two studies performed thus far which also used targeted capture enrichment for studying transcriptomes of *Candida* pathogens, namely *C. albicans* and *C. glabrata*, *in vivo* by RNA-Seq (Amorim-Vaz et al., 2015; Schrevens et al., 2022). Both studies used SureSelect enrichment technology (Agilent) targeting ORFomes of these species by non-overlapping oligo probes, and in the context of enrichment efficiency and fidelity both studies reported comparable results - the probes altered ~3-3.6% of targeted genes, and on average had ~1000 fold enrichment of fungal reads. Although fold-enrichment in our study, which used overlapping probes based on SeqCap technology, is significantly lower, this is likely related to the higher initial relative amount of fungal RNA in the studies of Amorim-Vaz et al. (2015) and Schrevens et al. (2022), as we found a relationship between this quantity and the resulting fold enrichment. Taking this into consideration, we show that both approaches have comparable efficiencies. Importantly, our probe set can potentially be extended to other probe-based enrichment technologies.

We show that our probe set has several clear advantages.

Firstly, our pan-*Candida* enrichment design not only targets the *C. albicans* transcriptome, but also includes probes to capture transcriptomes of three other major pathogens *C. glabrata*, *C. parapsilosis* and *C. tropicalis*. Although we have experimentally tested the kit for *C. albicans*, the probes were designed simultaneously for all four species in a uniform manner, thus it is unlikely that overall results of enrichment for other species will be drastically different from those of *C. albicans*. The benefit of this probe-mixing approach is two-fold. First, considering that the capacity of enrichment kits is usually far larger than the transcriptome of a single yeast, designing probes simultaneously for several species makes the enrichment kits more cost-effective, especially taking into account their high price. Second, apart from the possibility of analyzing several species, this design adds flexibility when performing enrichment experiments because it allows to mix several sequencing libraries together (which is usually necessary to reach a minimal volume required for efficient enrichment) containing different species.

Moreover, considering that the four *Candida* species are only distantly related despite their shared genus name, potential cross-hybridizations between probes and targets of different species can be effectively resolved bioinformatically on the read mapping step. In fact, *in-silico* analysis of read cross-mapping between the transcriptomes of four species demonstrates that paired-end sequencing with as short as 50 base pairs read length already results in practically no cross-mapping between reads for different species.

Secondly, our design includes the newly predicted lncRNAs of these four species, opening new avenues for studying the potential role of these enigmatic molecules in host-pathogen interactions between the human host and *Candida* yeasts.

Apart from thorough transcriptomic analysis, we asked whether the RNA-Seq data obtained after probe-based enrichment can serve for other types of analyses. Considering that RNA-Seq bears genotypic information of the transcribed regions of a genome, we assessed the feasibility of performing SNP calling analysis on our enriched data. Comparison between the pairs of non-enriched and enriched samples demonstrated that probe enrichment preserves 50-75% of original variants. Moreover, by comparing our genotyping data with those of other *C. albicans* strains, we show that it is possible to identify the infecting strain at a clade level. These results indicate that probe-enriched samples might additionally identify genotypic variants of the studied pathogen, which can serve as an important layer of information for fungal strain identification and antifungal susceptibility profiling.

## Conclusions

We successfully designed a pan-*Candida* probe-based enrichment approach using SeqCap technology, targeting the coding and non-coding transcriptomes of the four major *Candida* pathogens. We showed that this kit enriched fungal RNA more efficiently than the human cell lysis approach using large-scale dual RNA-Seq of human vaginal samples spiked with *C. albicans* cells. Moreover, we demonstrated that RNA-Seq data generated after probe enrichment can serve as a source for additional valuable analyses such as fungal genotyping. Our work highlights the power of targeted probe enrichment, which opens new horizons for investigating *in vivo* host-microbe interactions between human and *Candida* pathogens.

## Data availability statement

The data presented in the study are deposited in Sequence Read Archive (SRA, https://www.ncbi.nlm.nih.gov/sra) repository, accession number PRJNA721739. The data is now publicly release and publicly available.

## Ethics statement

The studies involving human participants were reviewed and approved by EC/2016/0192. The patients/participants provided their written informed consent to participate in this study.

## Author contributions

AR and ES performed the experiments. HH performed all computational analyses. TG and MV supervised the work and obtained funding. TG conceived the project. HH and TG wrote the first draft of the manuscript with contributions from all authors. All authors contributed to the article and approved the submitted version.

## Supplementary material

Supplementary Figure 1 The proportions of fungal lncRNA reads obtained with different enrichment approaches, as determined by RNA-Seq. Left panel shows data of experiments with 10^5^ C*. albicans* cells (“High fungal load”), right panel - with 10^3^ fungal cells (“Low fungal load”). Upper panel shows data on experiments with no probe-based enrichment, and the bottom panel - with probe-based enrichment. For each treatment N, T, B and BT (x axis), bars represent the proportion (in %, y axis) of mapped lncRNA fungal reads over the total number of mapped reads to *C. albicans* and the human genomes, calculated as mean and standard deviation across three replicates. Labels on the bars represent the mean percentage (at the top) and mean raw read counts (at the bottom) across replicates.

Supplementary Figure 2 Enrichment efficiency of probe-based enrichment (this study) compared to that of Amorim-Vaz et al. (2015). **(A)** Initial (*i.e*. before probe-based enrichment) and final (*i.e*. after probe-based enrichment) fungal proportions of the samples in both studies. Labels indicate the fold-enrichment - ratio between initial and final proportions; **(B)** Linear model of fold enrichment depending on the initial fungal proportions; **(C)** Comparison of fold enrichments observed in Amorim-Vaz et al. (2015), and predicted fold enrichment of our probes based on the linear model and initial fungal proportions reported in Amorim-Vaz et al. (2015).

Supplementary Figure 3 Scatter plots displaying log2 normalized counts before and after probe-based enrichment. **(A)** Data of the current study (for non pre-treated samples “N”). Top row - samples spiked with 10^5^ C*. albicans* cells; bottom row - samples spiked with 10^3^ C*. albicans* cells. Labels of the samples on axes are internal sample identifiers; **(B)** Data from Amorim-Vaz et al. (2015). Labels on the axes are sample labels from that study.

Supplementary Figure 4 Distribution of SNPs after probe-based enrichment compared to the non-enriched samples. The analysis compares enriched and corresponding non-enriched samples of each replicate. The comparison between samples of each replicate is shown by a dot. Colors of dots correspond to the replicate number. Box plots show the distribution across replicates. Sensitivity: proportion (in %) of the number of common variants between enriched and non-enriched samples divided by the number of variants in the non-enriched sample. Accuracy: proportion (in %) of the number of common variants between enriched and non-enriched samples divided by the number of variants in the probe-enriched sample.

Supplementary Figure 5 The Distribution of log2 read depth (DP) values of variant calling results. On the X axis, “Intersect” corresponds to log2(DP) values of variants identified in both probe-enriched and non-enriched samples (the values of non-enriched variant are plotted); “Only_non_enriched” corresponds to log2(DP) values of variants identified exclusively in non-enriched samples. N, T, B, BT and 1,2,3 correspond to host cells lysis method and replicates.

Supplementary Figure 6 Comparison of variants of enriched and non-enriched samples (treatment “N”) with sequenced *C. albicans* strains (52). Each bar represents the proportion obtained by dividing the number of variants in common between the strain used in this study and a given published strain by the total number of variants of the published strain. Only strains with top 11 proportions are plotted. **(A)** Results for 10^5^ fungal load; **(B)** Results for 10^3^ fungal load.

Supplementary Figure 7 The distribution (in %) of homozygous and heterozygous variants after probe-based enrichment compared to non-enriched samples. N, T, B, BT and 1,2,3 correspond to host cells lysis method and replicates.

Data Sheet 1 Read cross-mapping statistics between transcriptomes of the four studied species as assessed by Crossmapper software. The zipped file should be uncompressed and opened in any internet browser.
