# Supplementary figures and images for: Multiplexed target enrichment of coding and non-coding transcriptomes enables studying Candida spp. infections from human derived samples
Source: Front Cell Infect Microbiol. 2023 Jan 24;13:1093178. doi: 10.3389/fcimb.2023.1093178 (PMC9902369; doi:10.3389/fcimb.2023.1093178)

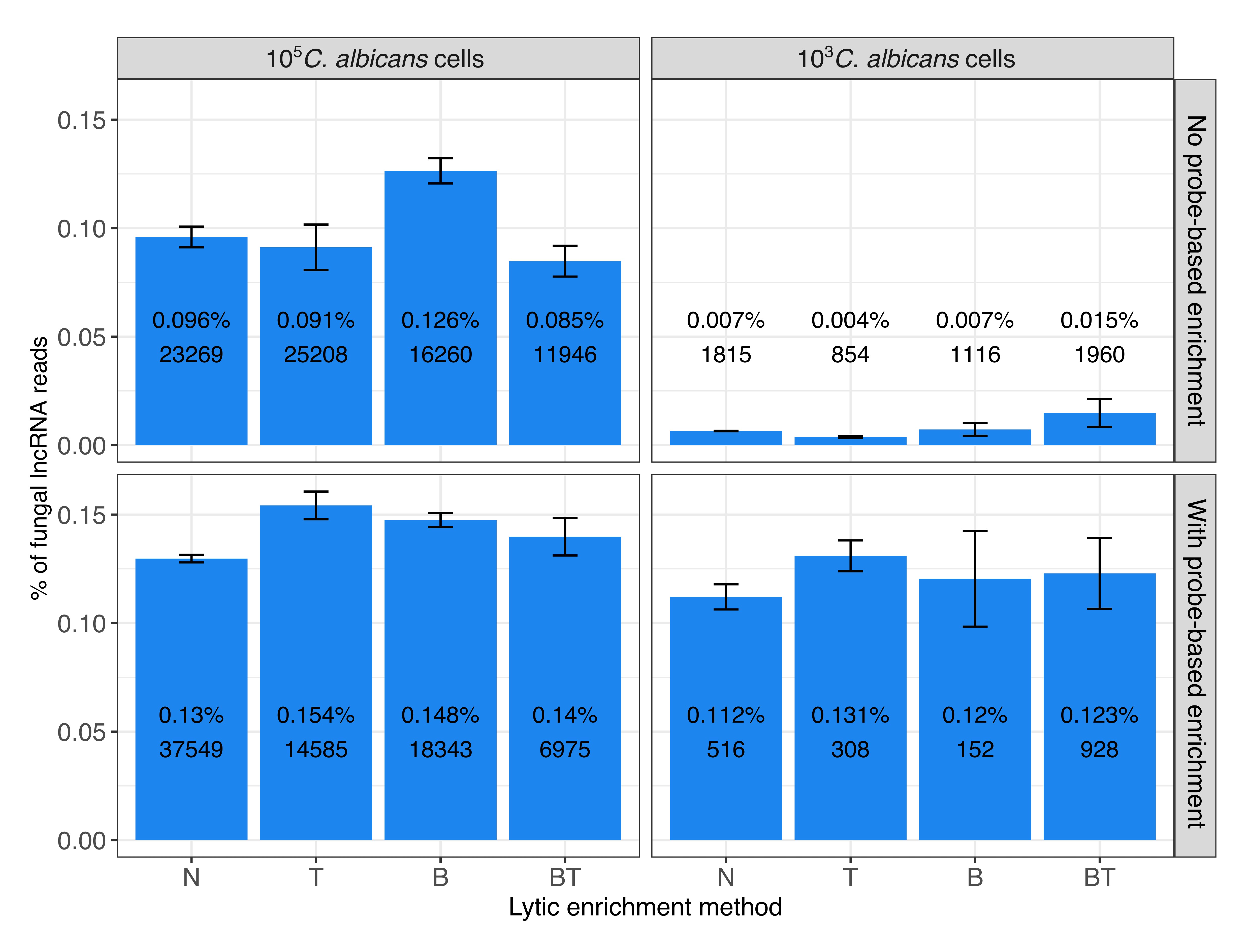

Supplement: Supplementary Figure 1 — The proportions of fungal lncRNA reads obtained with different enrichment approaches, as determined by RNA-Seq. Left panel shows data of experiments with 105 C. albicans cells (“High fungal load”), right panel - with 103 fungal cells (“Low fungal load”). Upper panel shows data on experiments with no probe-based enrichment, and the bottom panel - with probe-based enrichment. For each treatment N, T, B and BT (x axis), bars represent the proportion (in %, y axis) of mapped lncRNA fungal reads over the total number of mapped reads to C. albicans and the human genomes, calculated as mean and standard deviation across three replicates. Labels on the bars represent the mean percentage (at the top) and mean raw read counts (at the bottom) across replicates. [file Image_1.jpeg]

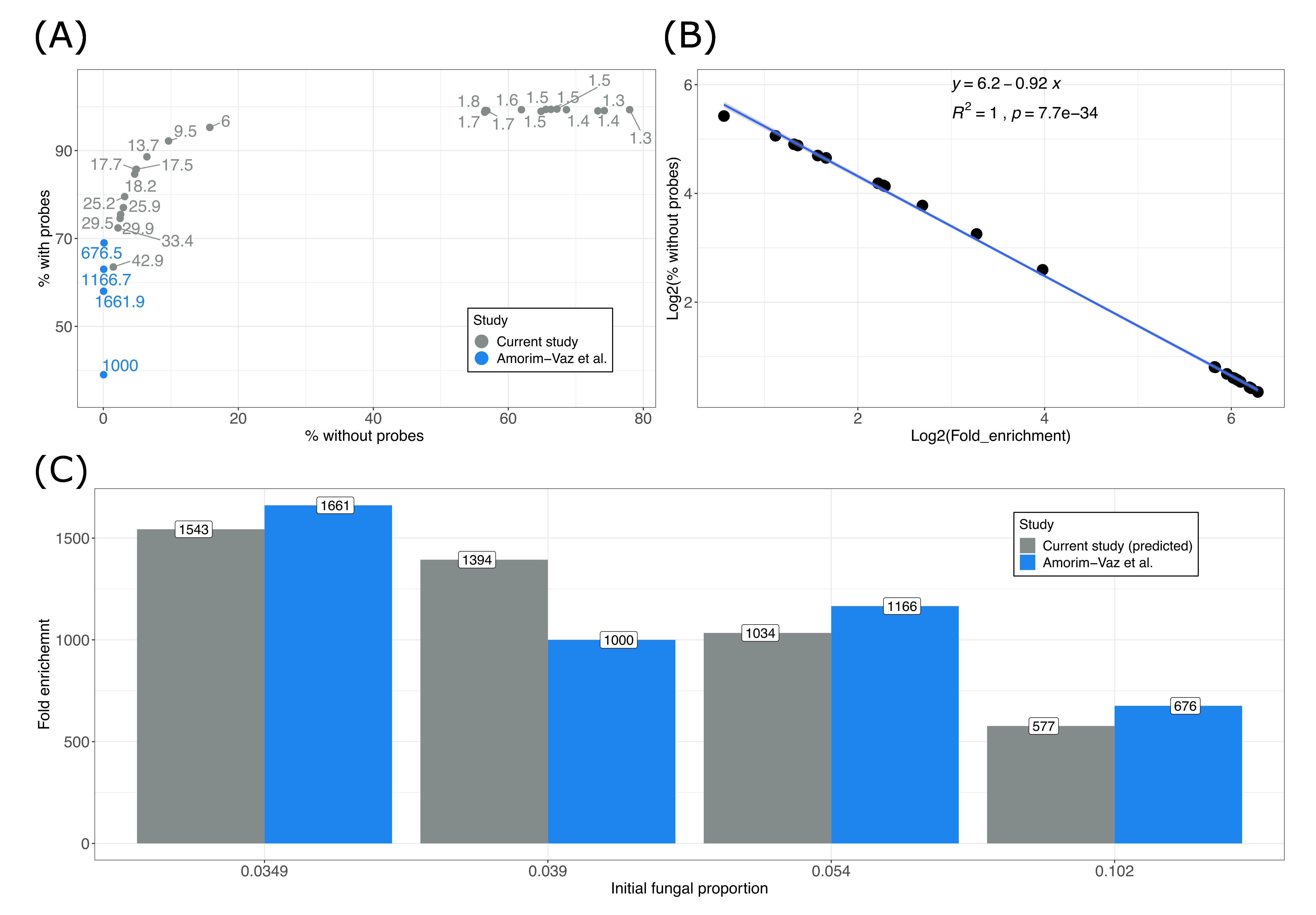

Supplement: Supplementary Figure 2 — Enrichment efficiency of probe-based enrichment (this study) compared to that of Amorim-Vaz et al. (2015). (A) Initial (i.e. before probe-based enrichment) and final (i.e. after probe-based enrichment) fungal proportions of the samples in both studies. Labels indicate the fold-enrichment - ratio between initial and final proportions; (B) Linear model of fold enrichment depending on the initial fungal proportions; (C) Comparison of fold enrichments observed in Amorim-Vaz et al. (2015), and predicted fold enrichment of our probes based on the linear model and initial fungal proportions reported in Amorim-Vaz et al. (2015). [file Image_2.jpeg]

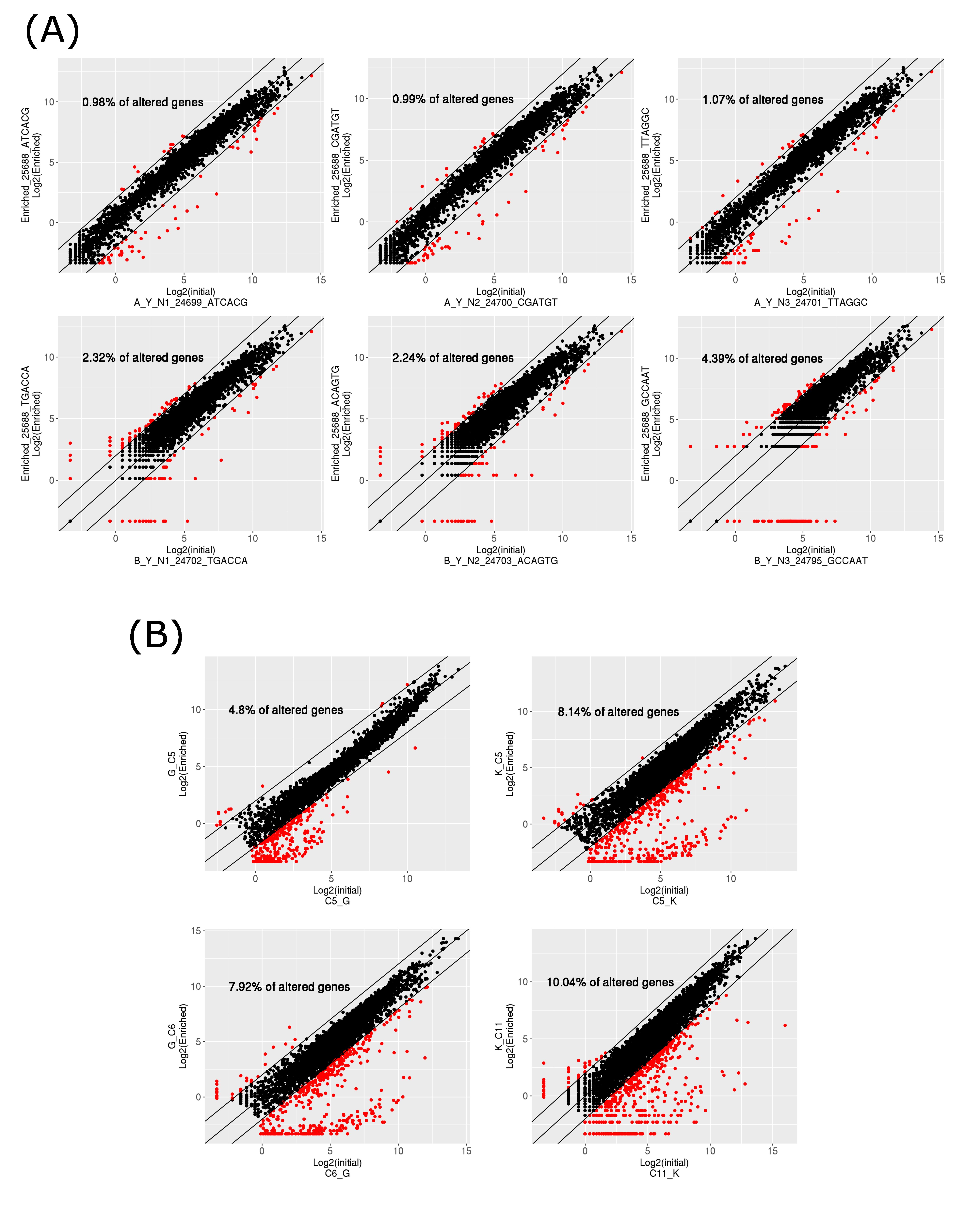

Supplement: Supplementary Figure 3 — Scatter plots displaying log2 normalized counts before and after probe-based enrichment. (A) Data of the current study (for non pre-treated samples “N”). Top row - samples spiked with 105 C. albicans cells; bottom row - samples spiked with 103 C. albicans cells. Labels of the samples on axes are internal sample identifiers; (B) Data from Amorim-Vaz et al. (2015). Labels on the axes are sample labels from that study. [file Image_3.jpeg]

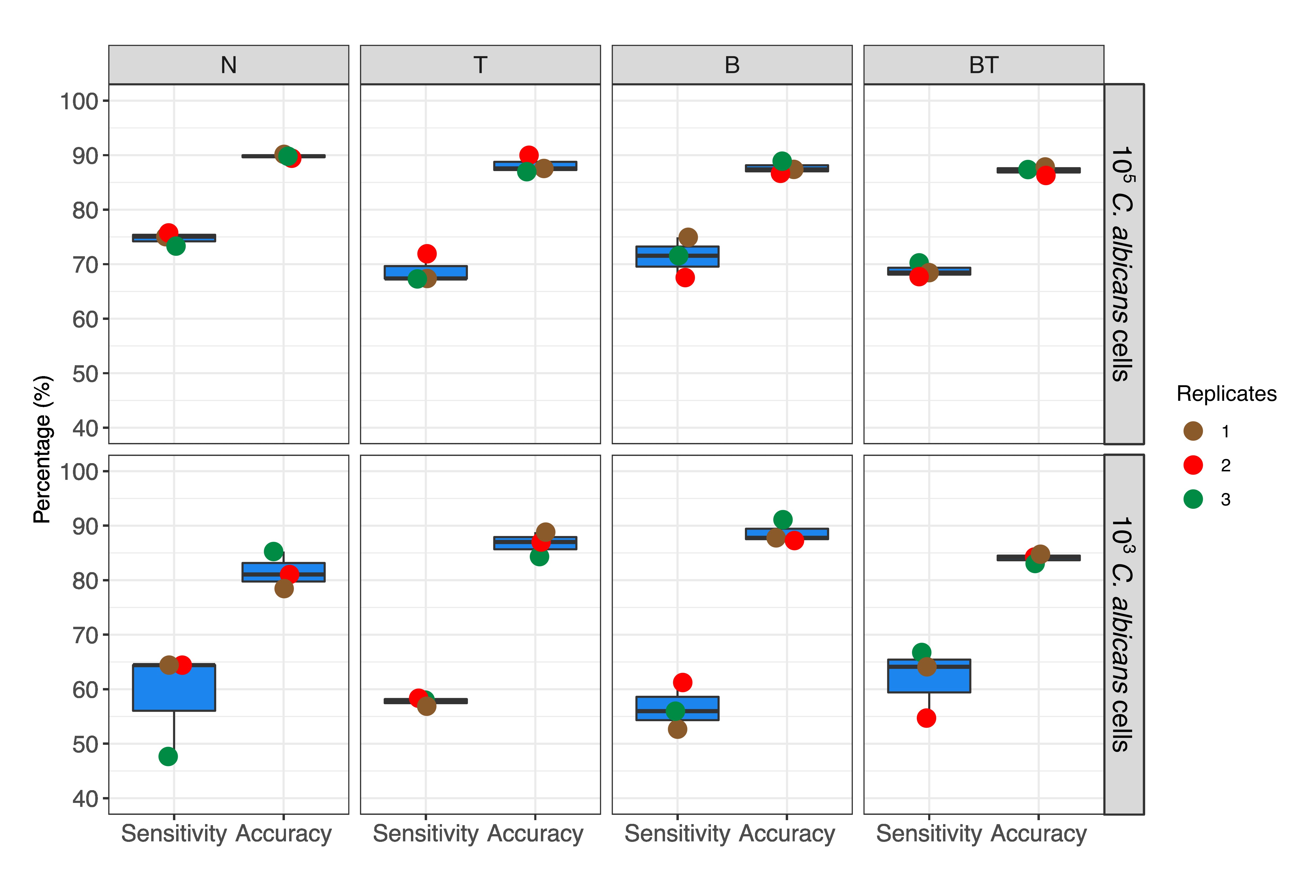

Supplement: Supplementary Figure 4 — Distribution of SNPs after probe-based enrichment compared to the non-enriched samples. The analysis compares enriched and corresponding non-enriched samples of each replicate. The comparison between samples of each replicate is shown by a dot. Colors of dots correspond to the replicate number. Box plots show the distribution across replicates. Sensitivity: proportion (in %) of the number of common variants between enriched and non-enriched samples divided by the number of variants in the non-enriched sample. Accuracy: proportion (in %) of the number of common variants between enriched and non-enriched samples divided by the number of variants in the probe-enriched sample. [file Image_4.jpeg]

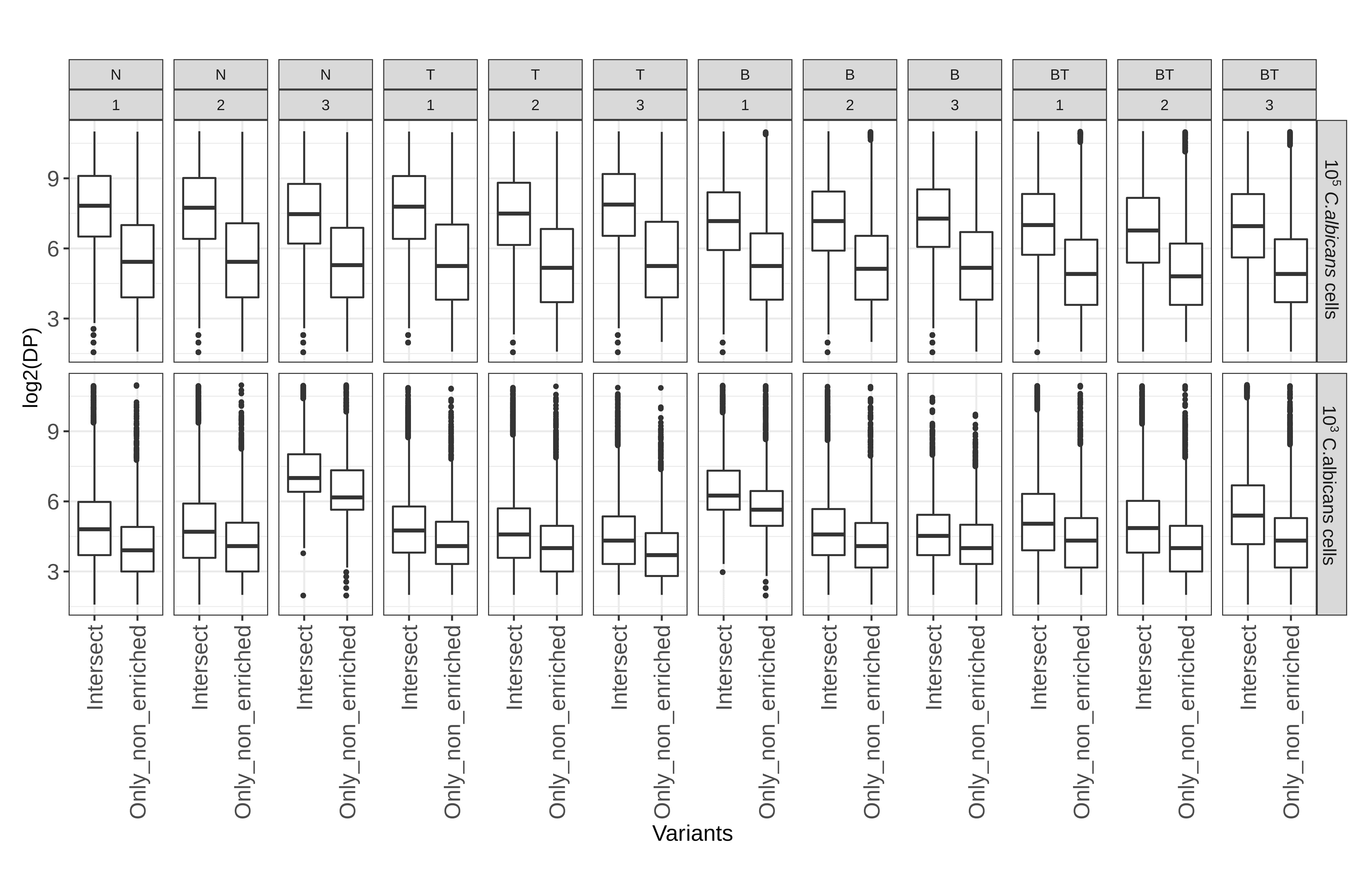

Supplement: Supplementary Figure 5 — The Distribution of log2 read depth (DP) values of variant calling results. On the X axis, “Intersect” corresponds to log2(DP) values of variants identified in both probe-enriched and non-enriched samples (the values of non-enriched variant are plotted); “Only_non_enriched” corresponds to log2(DP) values of variants identified exclusively in non-enriched samples. N, T, B, BT and 1,2,3 correspond to host cells lysis method and replicates. [file Image_5.jpeg]

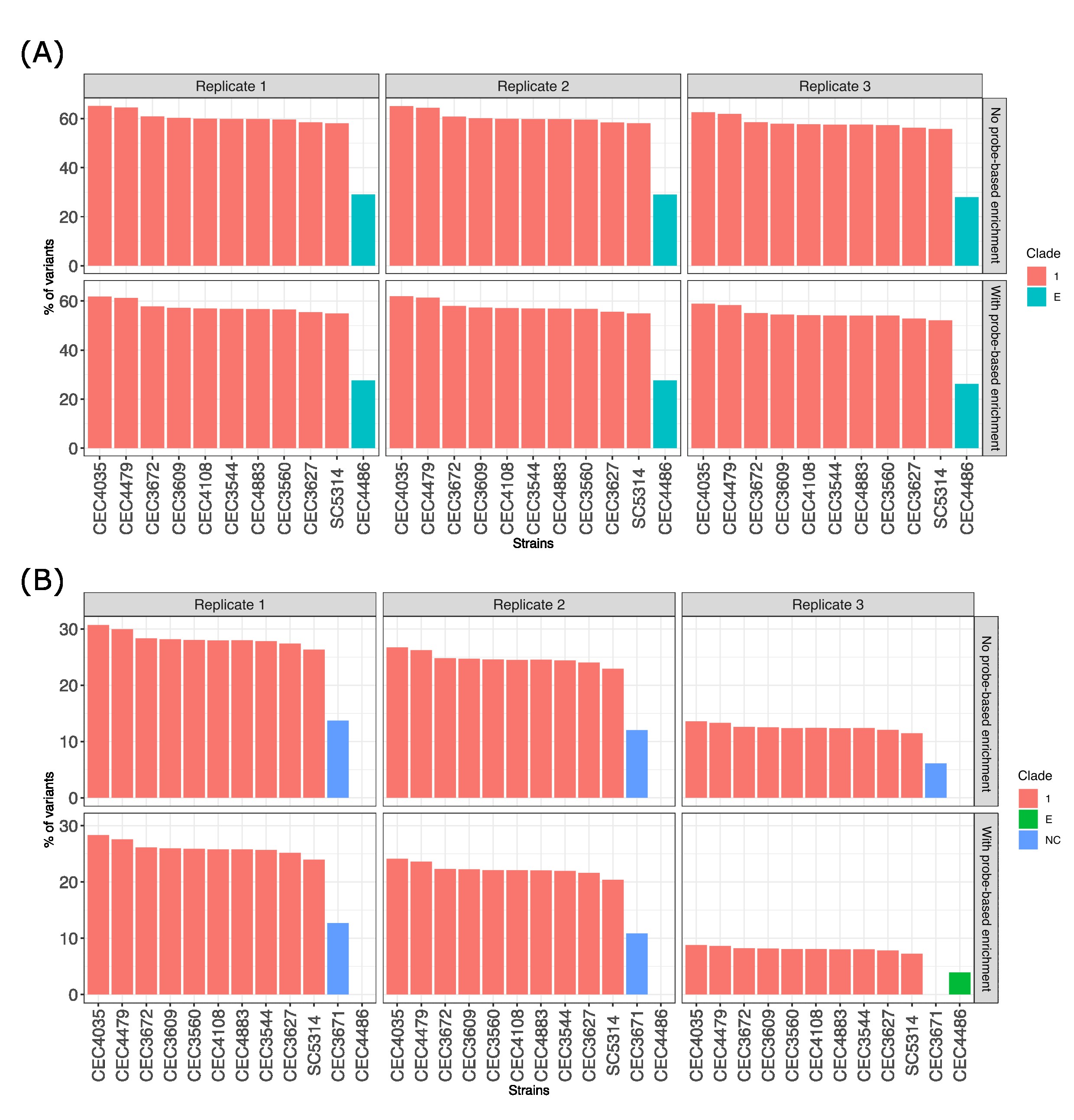

Supplement: Supplementary Figure 6 — Comparison of variants of enriched and non-enriched samples (treatment “N”) with sequenced C. albicans strains (52). Each bar represents the proportion obtained by dividing the number of variants in common between the strain used in this study and a given published strain by the total number of variants of the published strain. Only strains with top 11 proportions are plotted. (A) Results for 105 fungal load; (B) Results for 103 fungal load. [file Image_6.jpeg]

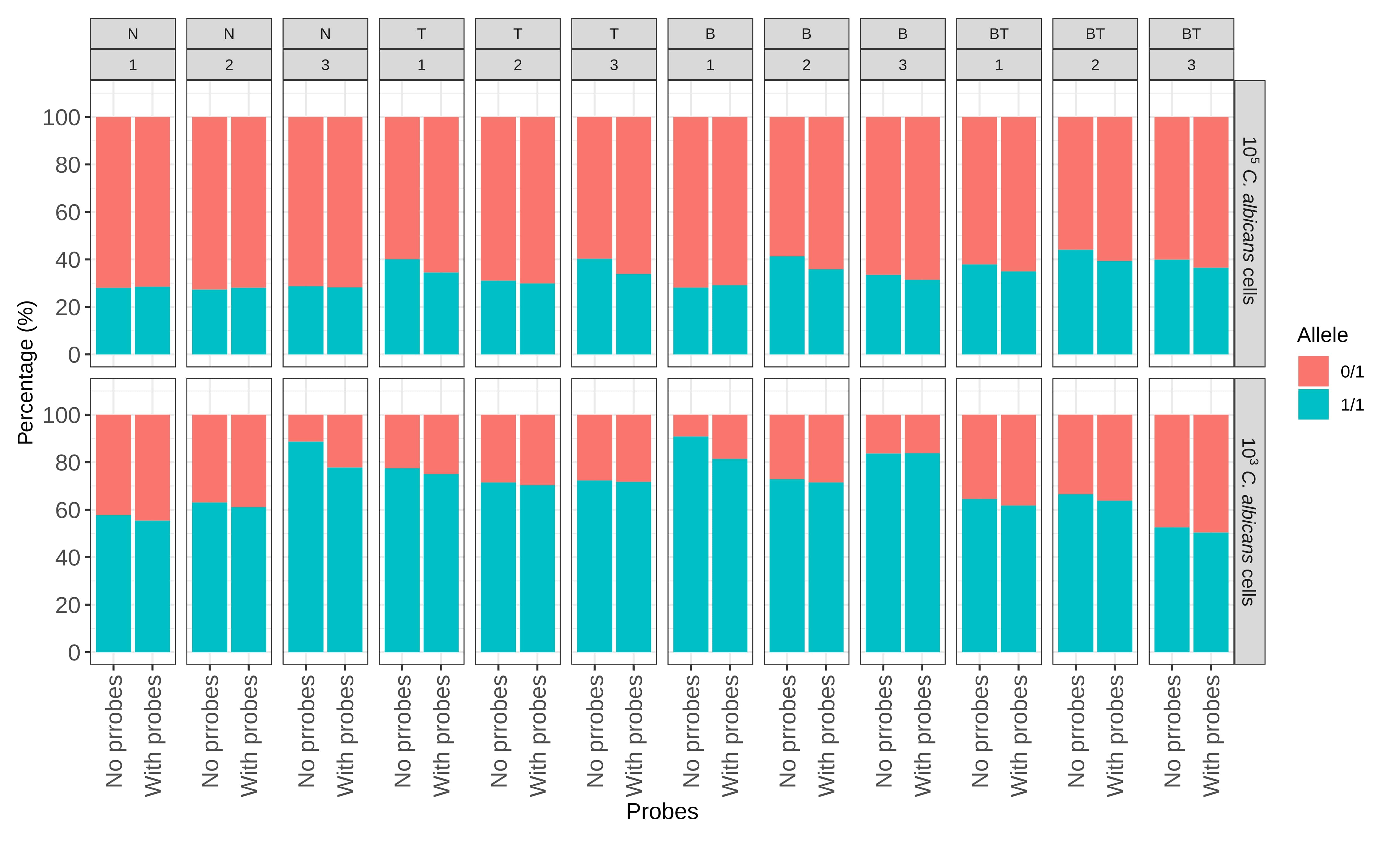

Supplement: Supplementary Figure 7 — The distribution (in %) of homozygous and heterozygous variants after probe-based enrichment compared to non-enriched samples. N, T, B, BT and 1,2,3 correspond to host cells lysis method and replicates. [file Image_7.jpeg]
