# Supplementary material for: Multiplexed target enrichment of coding and non-coding transcriptomes enables studying Candida spp. infections from human derived samples
Source: Front Cell Infect Microbiol. 2023 Jan 24;13:1093178. doi: 10.3389/fcimb.2023.1093178 (PMC9902369; doi:10.3389/fcimb.2023.1093178)
Supplement: Data Sheet 1 — Read cross-mapping statistics between transcriptomes of the four studied species as assessed by Crossmapper software. The zipped file should be uncompressed and opened in any internet browser. [file DataSheet_1.zip › File_S1.html]

Switch to

Crossmapper Summary Report

---

---

Mapping summary for read length 50 and SE layout


---

Overall mapping statistics

|  | #Reads | Percent |
| Uniquely Mapped Reads | 33694464 | 87.6% |
| Multimapped Reads | 3201238 | 8.32% |
| Unmapped Reads | 1569088 | 4.08% |
| Total Reads | 38464790 | -- |

  

Correct and Incorrect mapping statistics

| Genome | Correct | | Incorrect | | |
| Unique | Multi | Unique Cross | Multi | |
| Source and Other | Only Other |
| calb | 8632071 | 241387 | 354 | 214427 | 17 |
| ctrop | 8466644 | 447709 | 376 | 212119 | 19 |
| cpar | 8777935 | 328665 | 233 | 91896 | 26 |
| cglab | 7816800 | 1640714 | 51 | 24251 | 8 |

  

Breakdown of Crossmapping by organisms

|  | Unique Cross | | | | Multi Cross | | | | Total Cross | | | |
| calb | ctrop | cpar | cglab | calb | ctrop | cpar | cglab | calb | ctrop | cpar | cglab |
| calb | - | 248 | 88 | 18 | - | 177720 | 50257 | 12003 | - | 177968 | 50345 | 12021 |
| ctrop | 261 | - | 103 | 12 | 177140 | - | 46614 | 11504 | 177401 | - | 46717 | 11516 |
| cpar | 102 | 113 | - | 18 | 54156 | 51291 | - | 10942 | 54258 | 51404 | - | 10960 |
| cglab | 13 | 10 | 28 | - | 12351 | 10982 | 11280 | - | 12364 | 10992 | 11308 | - |

---

Mapping summary for read length 50 and PE layout


---

Overall mapping statistics

|  | #Reads | Percent |
| Uniquely Mapped Reads | 76799991 | 99.83% |
| Multimapped Reads | 1304 | 0.0% |
| Unmapped Reads | 128285 | 0.17% |
| Total Reads | 76929580 | -- |

  

Correct and Incorrect mapping statistics

| Genome | Correct | | Incorrect | | |
| Unique | Multi | Unique Cross | Multi | |
| Source and Other | Only Other |
| calb | 18916695 | 116 | 3068 | 115 | 0 |
| ctrop | 18997722 | 220 | 267 | 127 | 0 |
| cpar | 19143370 | 144 | 2991 | 87 | 1 |
| cglab | 19734747 | 480 | 1131 | 14 | 0 |

  

Breakdown of Crossmapping by organisms

|  | Unique Cross | | | | Multi Cross | | | | Total Cross | | | |
| calb | ctrop | cpar | cglab | calb | ctrop | cpar | cglab | calb | ctrop | cpar | cglab |
| calb | - | 209 | 2242 | 617 | - | 94 | 37 | 4 | - | 303 | 2279 | 621 |
| ctrop | 217 | - | 41 | 9 | 103 | - | 38 | 6 | 320 | - | 79 | 15 |
| cpar | 2258 | 20 | - | 713 | 57 | 66 | - | 14 | 2315 | 86 | - | 727 |
| cglab | 598 | 0 | 533 | - | 10 | 9 | 10 | - | 608 | 9 | 543 | - |

---

Mapping summary for read length 100 and SE layout


---

Overall mapping statistics

|  | #Reads | Percent |
| Uniquely Mapped Reads | 35026850 | 91.06% |
| Multimapped Reads | 3419520 | 8.89% |
| Unmapped Reads | 18420 | 0.05% |
| Total Reads | 38464790 | -- |

  

Correct and Incorrect mapping statistics

| Genome | Correct | | Incorrect | | |
| Unique | Multi | Unique Cross | Multi | |
| Source and Other | Only Other |
| calb | 8966658 | 272073 | 14 | 232805 | 0 |
| ctrop | 8786600 | 499435 | 20 | 224825 | 0 |
| cpar | 9138635 | 369912 | 10 | 76107 | 1 |
| cglab | 8134909 | 1728350 | 4 | 16012 | 0 |

  

Breakdown of Crossmapping by organisms

|  | Unique Cross | | | | Multi Cross | | | | Total Cross | | | |
| calb | ctrop | cpar | cglab | calb | ctrop | cpar | cglab | calb | ctrop | cpar | cglab |
| calb | - | 13 | 1 | 0 | - | 199767 | 35377 | 6074 | - | 199780 | 35378 | 6074 |
| ctrop | 14 | - | 4 | 2 | 195598 | - | 29823 | 4603 | 195612 | - | 29827 | 4605 |
| cpar | 3 | 6 | - | 1 | 42016 | 35975 | - | 6340 | 42019 | 35981 | - | 6341 |
| cglab | 2 | 1 | 1 | - | 7959 | 5559 | 7839 | - | 7961 | 5560 | 7840 | - |

---

Mapping summary for read length 100 and PE layout


---

Overall mapping statistics

|  | #Reads | Percent |
| Uniquely Mapped Reads | 76929506 | 100.0% |
| Multimapped Reads | 46 | 0.0% |
| Unmapped Reads | 28 | 0.0% |
| Total Reads | 76929580 | -- |

  

Correct and Incorrect mapping statistics

| Genome | Correct | | Incorrect | | |
| Unique | Multi | Unique Cross | Multi | |
| Source and Other | Only Other |
| calb | 18950565 | 0 | 1506 | 1 | 0 |
| ctrop | 19030858 | 7 | 0 | 1 | 0 |
| cpar | 19177038 | 15 | 1476 | 9 | 0 |
| cglab | 19767659 | 13 | 404 | 0 | 0 |

  

Breakdown of Crossmapping by organisms

|  | Unique Cross | | | | Multi Cross | | | | Total Cross | | | |
| calb | ctrop | cpar | cglab | calb | ctrop | cpar | cglab | calb | ctrop | cpar | cglab |
| calb | - | 0 | 1348 | 158 | - | 1 | 1 | 0 | - | 1 | 1349 | 158 |
| ctrop | 0 | - | 0 | 0 | 1 | - | 0 | 0 | 1 | - | 0 | 0 |
| cpar | 1246 | 0 | - | 230 | 9 | 9 | - | 2 | 1255 | 9 | - | 232 |
| cglab | 182 | 0 | 222 | - | 0 | 0 | 0 | - | 182 | 0 | 222 | - |

---

Mapping summary for read length 150 and SE layout


---

Overall mapping statistics

|  | #Reads | Percent |
| Uniquely Mapped Reads | 35291450 | 91.75% |
| Multimapped Reads | 3173120 | 8.25% |
| Unmapped Reads | 220 | 0.0% |
| Total Reads | 38464790 | -- |

  

Correct and Incorrect mapping statistics

| Genome | Correct | | Incorrect | | |
| Unique | Multi | Unique Cross | Multi | |
| Source and Other | Only Other |
| calb | 9055999 | 264924 | 1 | 155046 | 0 |
| ctrop | 8878139 | 490502 | 0 | 146749 | 0 |
| cpar | 9187279 | 358944 | 0 | 43002 | 0 |
| cglab | 8170032 | 1705536 | 0 | 8417 | 0 |

  

Breakdown of Crossmapping by organisms

|  | Unique Cross | | | | Multi Cross | | | | Total Cross | | | |
| calb | ctrop | cpar | cglab | calb | ctrop | cpar | cglab | calb | ctrop | cpar | cglab |
| calb | - | 0 | 1 | 0 | - | 136817 | 19209 | 3906 | - | 136817 | 19210 | 3906 |
| ctrop | 0 | - | 0 | 0 | 132580 | - | 14225 | 1279 | 132580 | - | 14225 | 1279 |
| cpar | 0 | 0 | - | 0 | 23987 | 19374 | - | 4141 | 23987 | 19374 | - | 4141 |
| cglab | 0 | 0 | 0 | - | 5299 | 2015 | 5366 | - | 5299 | 2015 | 5366 | - |

---

Mapping summary for read length 150 and PE layout


---

Overall mapping statistics

|  | #Reads | Percent |
| Uniquely Mapped Reads | 76929537 | 100.0% |
| Multimapped Reads | 49 | 0.0% |
| Unmapped Reads | 0 | 0.0% |
| Total Reads | 76929586 | -- |

  

Correct and Incorrect mapping statistics

| Genome | Correct | | Incorrect | | |
| Unique | Multi | Unique Cross | Multi | |
| Source and Other | Only Other |
| calb | 18951451 | 2 | 621 | 2 | 0 |
| ctrop | 19030857 | 16 | 0 | 1 | 0 |
| cpar | 19177958 | 15 | 578 | 3 | 0 |
| cglab | 19768028 | 10 | 44 | 0 | 0 |

  

Breakdown of Crossmapping by organisms

|  | Unique Cross | | | | Multi Cross | | | | Total Cross | | | |
| calb | ctrop | cpar | cglab | calb | ctrop | cpar | cglab | calb | ctrop | cpar | cglab |
| calb | - | 1 | 602 | 18 | - | 0 | 2 | 2 | - | 1 | 604 | 20 |
| ctrop | 0 | - | 0 | 0 | 0 | - | 1 | 0 | 0 | - | 1 | 0 |
| cpar | 562 | 0 | - | 16 | 3 | 3 | - | 0 | 565 | 3 | - | 16 |
| cglab | 16 | 0 | 28 | - | 0 | 0 | 0 | - | 16 | 0 | 28 | - |

---

Mapping summary for read length 50 and SE layout


---

Overall mapping statistics

|  | #Reads | Percent |
| Uniquely Mapped Reads | 33694464 | 87.6% |
| Multimapped Reads | 3201238 | 8.32% |
| Unmapped Reads | 1569088 | 4.08% |
| Total Reads | 38464790 | -- |

  

Correct and Incorrect mapping statistics

| Genome | Correct | | Incorrect | | |
| Unique | Multi | Unique Cross | Multi | |
| Source and Other | Only Other |
| calb | 22.44% | 0.63% | 0.0% | 0.56% | 0.0% |
| ctrop | 22.01% | 1.16% | 0.0% | 0.55% | 0.0% |
| cpar | 22.82% | 0.85% | 0.0% | 0.24% | 0.0% |
| cglab | 20.32% | 4.27% | 0.0% | 0.06% | 0.0% |

  

Breakdown of Crossmapping by organisms

|  | Unique Cross | | | | Multi Cross | | | | Total Cross | | | |
| calb | ctrop | cpar | cglab | calb | ctrop | cpar | cglab | calb | ctrop | cpar | cglab |
| calb | - | 0.001% | 0.0% | 0.0% | - | 0.482% | 0.136% | 0.033% | - | 0.482% | 0.136% | 0.033% |
| ctrop | 0.001% | - | 0.0% | 0.0% | 0.48% | - | 0.126% | 0.031% | 0.481% | - | 0.127% | 0.031% |
| cpar | 0.0% | 0.0% | - | 0.0% | 0.147% | 0.139% | - | 0.03% | 0.147% | 0.139% | - | 0.03% |
| cglab | 0.0% | 0.0% | 0.0% | - | 0.033% | 0.03% | 0.031% | - | 0.034% | 0.03% | 0.031% | - |

---

Mapping summary for read length 50 and PE layout


---

Overall mapping statistics

|  | #Reads | Percent |
| Uniquely Mapped Reads | 76799991 | 99.83% |
| Multimapped Reads | 1304 | 0.0% |
| Unmapped Reads | 128285 | 0.17% |
| Total Reads | 76929580 | -- |

  

Correct and Incorrect mapping statistics

| Genome | Correct | | Incorrect | | |
| Unique | Multi | Unique Cross | Multi | |
| Source and Other | Only Other |
| calb | 24.59% | 0.0% | 0.0% | 0.0% | 0.0% |
| ctrop | 24.69% | 0.0% | 0.0% | 0.0% | 0.0% |
| cpar | 24.88% | 0.0% | 0.0% | 0.0% | 0.0% |
| cglab | 25.65% | 0.0% | 0.0% | 0.0% | 0.0% |

  

Breakdown of Crossmapping by organisms

|  | Unique Cross | | | | Multi Cross | | | | Total Cross | | | |
| calb | ctrop | cpar | cglab | calb | ctrop | cpar | cglab | calb | ctrop | cpar | cglab |
| calb | - | 0.0% | 0.003% | 0.001% | - | 0.0% | 0.0% | 0.0% | - | 0.0% | 0.003% | 0.001% |
| ctrop | 0.0% | - | 0.0% | 0.0% | 0.0% | - | 0.0% | 0.0% | 0.0% | - | 0.0% | 0.0% |
| cpar | 0.003% | 0.0% | - | 0.001% | 0.0% | 0.0% | - | 0.0% | 0.003% | 0.0% | - | 0.001% |
| cglab | 0.001% | 0.0% | 0.001% | - | 0.0% | 0.0% | 0.0% | - | 0.001% | 0.0% | 0.001% | - |

---

Mapping summary for read length 100 and SE layout


---

Overall mapping statistics

|  | #Reads | Percent |
| Uniquely Mapped Reads | 35026850 | 91.06% |
| Multimapped Reads | 3419520 | 8.89% |
| Unmapped Reads | 18420 | 0.05% |
| Total Reads | 38464790 | -- |

  

Correct and Incorrect mapping statistics

| Genome | Correct | | Incorrect | | |
| Unique | Multi | Unique Cross | Multi | |
| Source and Other | Only Other |
| calb | 23.31% | 0.71% | 0.0% | 0.61% | 0.0% |
| ctrop | 22.84% | 1.3% | 0.0% | 0.58% | 0.0% |
| cpar | 23.76% | 0.96% | 0.0% | 0.2% | 0.0% |
| cglab | 21.15% | 4.49% | 0.0% | 0.04% | 0.0% |

  

Breakdown of Crossmapping by organisms

|  | Unique Cross | | | | Multi Cross | | | | Total Cross | | | |
| calb | ctrop | cpar | cglab | calb | ctrop | cpar | cglab | calb | ctrop | cpar | cglab |
| calb | - | 0.0% | 0.0% | 0.0% | - | 0.52% | 0.092% | 0.016% | - | 0.52% | 0.092% | 0.016% |
| ctrop | 0.0% | - | 0.0% | 0.0% | 0.509% | - | 0.078% | 0.012% | 0.509% | - | 0.078% | 0.012% |
| cpar | 0.0% | 0.0% | - | 0.0% | 0.109% | 0.094% | - | 0.016% | 0.109% | 0.094% | - | 0.016% |
| cglab | 0.0% | 0.0% | 0.0% | - | 0.021% | 0.014% | 0.02% | - | 0.021% | 0.014% | 0.02% | - |

---

Mapping summary for read length 100 and PE layout


---

Overall mapping statistics

|  | #Reads | Percent |
| Uniquely Mapped Reads | 76929506 | 100.0% |
| Multimapped Reads | 46 | 0.0% |
| Unmapped Reads | 28 | 0.0% |
| Total Reads | 76929580 | -- |

  

Correct and Incorrect mapping statistics

| Genome | Correct | | Incorrect | | |
| Unique | Multi | Unique Cross | Multi | |
| Source and Other | Only Other |
| calb | 24.63% | 0.0% | 0.0% | 0.0% | 0.0% |
| ctrop | 24.74% | 0.0% | 0.0% | 0.0% | 0.0% |
| cpar | 24.93% | 0.0% | 0.0% | 0.0% | 0.0% |
| cglab | 25.7% | 0.0% | 0.0% | 0.0% | 0.0% |

  

Breakdown of Crossmapping by organisms

|  | Unique Cross | | | | Multi Cross | | | | Total Cross | | | |
| calb | ctrop | cpar | cglab | calb | ctrop | cpar | cglab | calb | ctrop | cpar | cglab |
| calb | - | 0.0% | 0.002% | 0.0% | - | 0.0% | 0.0% | 0.0% | - | 0.0% | 0.002% | 0.0% |
| ctrop | 0.0% | - | 0.0% | 0.0% | 0.0% | - | 0.0% | 0.0% | 0.0% | - | 0.0% | 0.0% |
| cpar | 0.002% | 0.0% | - | 0.0% | 0.0% | 0.0% | - | 0.0% | 0.002% | 0.0% | - | 0.0% |
| cglab | 0.0% | 0.0% | 0.0% | - | 0.0% | 0.0% | 0.0% | - | 0.0% | 0.0% | 0.0% | - |

---

Mapping summary for read length 150 and SE layout


---

Overall mapping statistics

|  | #Reads | Percent |
| Uniquely Mapped Reads | 35291450 | 91.75% |
| Multimapped Reads | 3173120 | 8.25% |
| Unmapped Reads | 220 | 0.0% |
| Total Reads | 38464790 | -- |

  

Correct and Incorrect mapping statistics

| Genome | Correct | | Incorrect | | |
| Unique | Multi | Unique Cross | Multi | |
| Source and Other | Only Other |
| calb | 23.54% | 0.69% | 0.0% | 0.4% | 0.0% |
| ctrop | 23.08% | 1.28% | 0.0% | 0.38% | 0.0% |
| cpar | 23.88% | 0.93% | 0.0% | 0.11% | 0.0% |
| cglab | 21.24% | 4.43% | 0.0% | 0.02% | 0.0% |

  

Breakdown of Crossmapping by organisms

|  | Unique Cross | | | | Multi Cross | | | | Total Cross | | | |
| calb | ctrop | cpar | cglab | calb | ctrop | cpar | cglab | calb | ctrop | cpar | cglab |
| calb | - | 0.0% | 0.0% | 0.0% | - | 0.356% | 0.05% | 0.01% | - | 0.356% | 0.05% | 0.01% |
| ctrop | 0.0% | - | 0.0% | 0.0% | 0.345% | - | 0.037% | 0.003% | 0.345% | - | 0.037% | 0.003% |
| cpar | 0.0% | 0.0% | - | 0.0% | 0.062% | 0.05% | - | 0.011% | 0.062% | 0.05% | - | 0.011% |
| cglab | 0.0% | 0.0% | 0.0% | - | 0.014% | 0.005% | 0.014% | - | 0.014% | 0.005% | 0.014% | - |

---

Mapping summary for read length 150 and PE layout


---

Overall mapping statistics

|  | #Reads | Percent |
| Uniquely Mapped Reads | 76929537 | 100.0% |
| Multimapped Reads | 49 | 0.0% |
| Unmapped Reads | 0 | 0.0% |
| Total Reads | 76929586 | -- |

  

Correct and Incorrect mapping statistics

| Genome | Correct | | Incorrect | | |
| Unique | Multi | Unique Cross | Multi | |
| Source and Other | Only Other |
| calb | 24.63% | 0.0% | 0.0% | 0.0% | 0.0% |
| ctrop | 24.74% | 0.0% | 0.0% | 0.0% | 0.0% |
| cpar | 24.93% | 0.0% | 0.0% | 0.0% | 0.0% |
| cglab | 25.7% | 0.0% | 0.0% | 0.0% | 0.0% |

  

Breakdown of Crossmapping by organisms

|  | Unique Cross | | | | Multi Cross | | | | Total Cross | | | |
| calb | ctrop | cpar | cglab | calb | ctrop | cpar | cglab | calb | ctrop | cpar | cglab |
| calb | - | 0.0% | 0.001% | 0.0% | - | 0.0% | 0.0% | 0.0% | - | 0.0% | 0.001% | 0.0% |
| ctrop | 0.0% | - | 0.0% | 0.0% | 0.0% | - | 0.0% | 0.0% | 0.0% | - | 0.0% | 0.0% |
| cpar | 0.001% | 0.0% | - | 0.0% | 0.0% | 0.0% | - | 0.0% | 0.001% | 0.0% | - | 0.0% |
| cglab | 0.0% | 0.0% | 0.0% | - | 0.0% | 0.0% | 0.0% | - | 0.0% | 0.0% | 0.0% | - |

---
